# Protective Effect of Resveratrol in an Experimental Model of Salicylate-Induced Tinnitus

**DOI:** 10.3390/ijms232214183

**Published:** 2022-11-16

**Authors:** Anji Song, Gwang-Won Cho, Changjong Moon, Ilyong Park, Chul Ho Jang

**Affiliations:** 1 Department of Biology, College of Natural Science, Chosun University, Gwangju 61452, Republic of Korea; dkswl511@naver.com (A.S.); gwcho@chosun.ac.kr (G.-W.C.); 2 BK21 FOUR Education Research Group for Age-Associated Disorder Control Technology, Department of Integrative Biological Science, Chosun University, Gwangju 61452, Republic of Korea; 3 Department of Veterinary Anatomy, College of Veterinary Medicine and BK21 FOUR Program, Chonnam Natioanal University, Gwanjgu 61186, Republic of Korea; moonc@chonnam.ac.kr; 4 Department of Biomedical Engineering, College of Medicine, Dankook University, Cheonan 31116, Republic of Korea; piyong@dankook.ac.kr; 5 Department of Otolaryngology, Chonnam National University Medical School, Gwangju 61469, Republic of Korea

**Keywords:** resveratrol, NMDA receptor, salicylate, excitotoxicity, tinnitus

## Abstract

To date, the effect of resveratrol on tinnitus has not been reported. The attenuative effects of resveratrol (RSV) on a salicylate-induced tinnitus model were evaluated by in vitro and in vivo experiments. The gene expression of the activity-regulated cytoskeleton-associated protein (*ARC*), tumor necrosis factor-alpha (*TNFα*), and NMDA receptor subunit 2B (*NR2B*) in SH-SY5Y cells was examined using qPCR. Phosphorylated cAMP response element-binding protein (p-CREB), apoptosis markers, and reactive oxygen species (ROS) were evaluated by in vitro experiments. The in vivo experiment evaluated the gap-prepulse inhibition of the acoustic startle reflex (GPIAS) and auditory brainstem response (ABR) level. The *NR2B* expression in the auditory cortex (AC) was determined by immunohistochemistry. RSV significantly reduced the salicylate-induced expression of *NR2B*, *ARC*, and *TNFα* in neuronal cells; the GPIAS and ABR thresholds altered by salicylate in rats were recovered close to their normal range. RSV also reduced the salicylate-induced NR2B overexpression of the AC. These results confirmed that resveratrol exerted an attenuative effect on salicylate-induced tinnitus and may have a therapeutic potential.

## 1. Introduction

Tinnitus is the conscious perception of sound without any corresponding external acoustic stimulus, commonly described as a phantom sound [1]. In many cases, tinnitus is caused by damage to the cochlear or auditory nerve or the central auditory pathway [2]. It is accompanied by symptoms such as anxiety, emotional disturbances, sleep disturbances, and work disorders, among others [1,3]. So far, there is no FDA-approved treatment for tinnitus. Although tinnitus can be transiently suppressed by lidocaine, no drugs have been shown to reverse the neural hyperactivity at the root of tinnitus. Existing medications cannot cure tinnitus, but can relieve a few of the serious symptoms of tinnitus.

Salicylate, one of the most widely used drugs, is prescribed for treating pain, headaches, a high fever, and inflammation. Nowadays, salicylate is commonly used in the secondary prevention of chronic coronary syndromes. However, its long-term use can cause tinnitus and eventually hearing loss due to an auditory nerve dysfunction [4,5].

A consequential nerve malfunction is the result of abnormal excitability at the brainstem and cerebral cortex [6], which is induced by the overactivity of the N-methyl-D-aspartate (NMDA) receptor subunit 2B (NR2B) [7,8,9,10]. Several studies have suggested that an increase in the expression of NMDA receptors causes ototoxicity in the cochlea [11,12]. A high dose of salicylate inhibits cyclooxygenase (COX) activity and increases the level of arachidonic acid in the lipid bilayers, which increases the open probability of NMDA receptors [10].

Resveratrol (RSV) is a polyphenol substance contained in most berries. It is known to have antioxidant, anti-aging, and anti-inflammatory effects [13]. RSV also mediates neuroprotective effects by maintaining the expression of glutamatergic and cholinergic receptors via influencing the sirtuin1 (SIRT1) expression [14]. The protective effect of RSV against cisplatin-induced ototoxicity using the HEI-OC1 cell line via antioxidants has been reported [15]. Although the neuroprotective role of RSV is well-reported, there are no reports on the attenuative effect of RSV on salicylate- or noise-induced tinnitus. In this study, we evaluated the protective effect of RSV in salicylate-induced in vitro and in vivo models. 

## 2. Results

### 2.1. RSV Induces a Decrease in the NR2B Expression in Neuronal Cells

**Figure 1:**
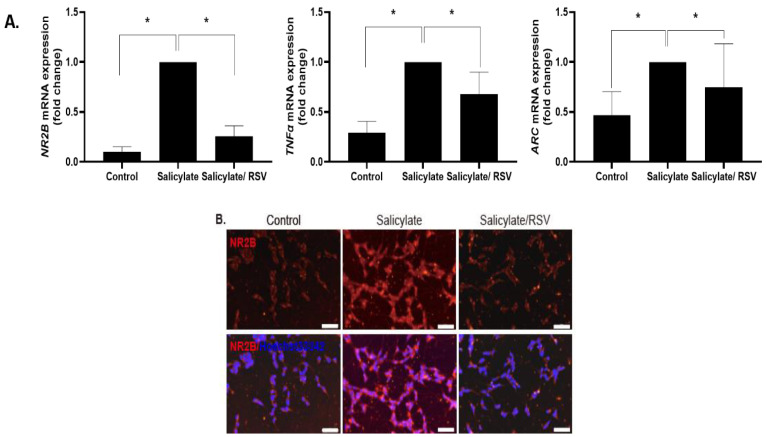

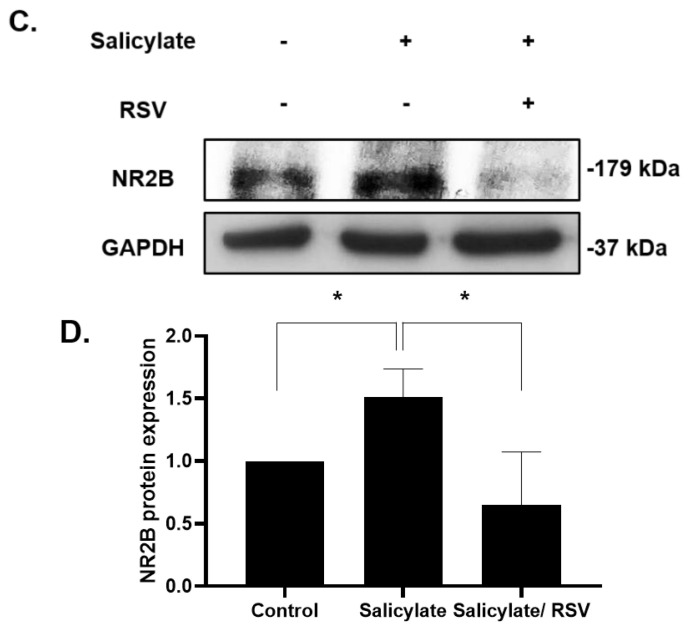
Expression of NR2B, *TNFα*, and the immediate early gene *ARC* in RSV-treated neuronal cells. Salicylate significantly increased the expression of *NR2B*, *TNFα*, and the immediate early gene *ARC,* whose expression was decreased in resveratrol (RSV) pretreated SH-SY5Y cells, as measured by quantitative polymerase chain reaction (**A**); * *p* < 0.05, mean ± SD. NR2B expression in SH-SY5Y cells increased with salicylate treatment and decreased with RSV pretreatment, as demonstrated by immunocytochemical staining (**B**) and immunoblot analysis (**C**). Western blot experiments were repeated and the bands were quantified using ImageJ software (**D**); * *p* < 0.05, mean ± SD. ARC: activity-regulated cytoskeleton-associated protein; TNFα: tumor necrosis factor-alpha; NR2B: NMDA receptor subunit 2B. Scale bar = 50 um.

SH-SY5Y cells were differentiated into neuron-like cells and treated with RSV or salicylate. The expression of *NR2B*, *ARC*, and *TNFα* increased in the salicylate-treated cells, but decreased in the RSV-pretreated cells, as measured by qPCR (Figure 1A). The NR2B expression was determined by immunocytochemical staining and an immunoblot analysis; consistent results were obtained (Figure 1B–D).

### 2.2. RSV Pretreatment Decreases Phosphorylated CREB in Salicylate-Treated Neuronal Cells

**Figure 2:**
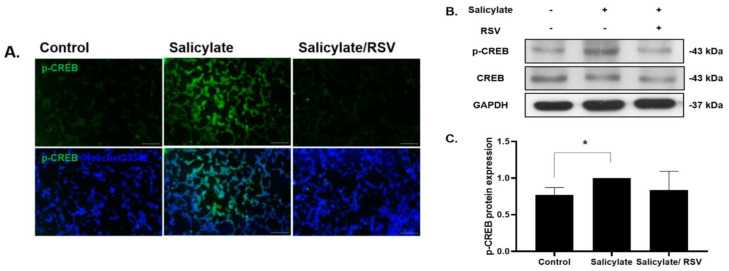
Phosphorylation of cAMP response element-binding protein (CREB) was reduced in resveratrol (RSV)-pretreated neuronal cells. Salicylate treatment induced CREB phosphorylation, which was reduced in RSV-pretreated SH-SY5Y cells as shown by immunocytochemistry staining (**A**) (scale bar = 100 µm) and immunoblot analysis (**B**). Western blot data were quantified and presented as a graph (**C**); * *p* < 0.05, mean ± SD. GAPDH was used as the internal standard. Scale bar = 100 µm.

Immunocytochemical staining and an immunoblotting analysis demonstrated that the p-CREB content increased in the salicylate-treated cells. There was no decrease with the RSV pretreatment (Figure 2A,B). Immunoblot assays were repeated three times and the p-CREB expression was quantified (Figure 2C).

### 2.3. RSV Inhibits a ROS Increase in Salicylate-Treated Neuronal Cells

**Figure 3:**
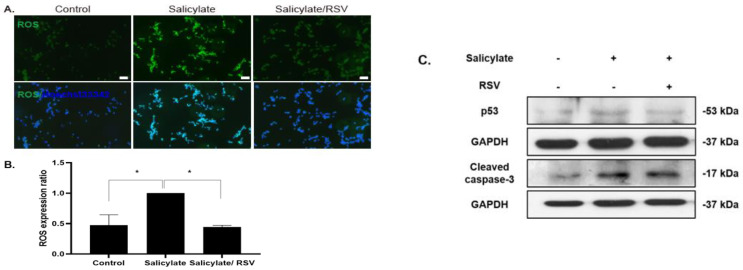
Pretreatment of resveratrol (RSV) reduced intracellular reactive oxygen species (ROS) levels in salicylate-treated neuronal cells. ROS levels increased with salicylate treatment, but decreased in RSV-pretreated neuronal cells as observed by fluorescence microscopy (**A**). The ROS levels were quantified using ImageJ (**B**); * *p* < 0.05, mean ± SD. Salicylate increased the expression of cleaved caspase-3 and p53, which was deceased in RSV-pretreated cells (**C**); scale bar = 100 µm. GAPDH was used as the internal standard. Scale bar = 100 µm.

To determine the intracellular ROS levels in salicylate-treated neuronal cells, ROS-detecting fluorescent DCFH-DA was used. The ROS levels increased in the salicylate-treated cells, but decreased with the RSV pretreatment (Figure 3A,B). The immunoblot analysis revealed that the salicylate-induced increase in p53 and cleaved caspase-3 expression was reduced when the neuronal cells were pretreated with RSV (Figure 3C).

### 2.4. Effect of RSV on Salicylate-Treated Rat Cortical Neurons

**Figure 4:**
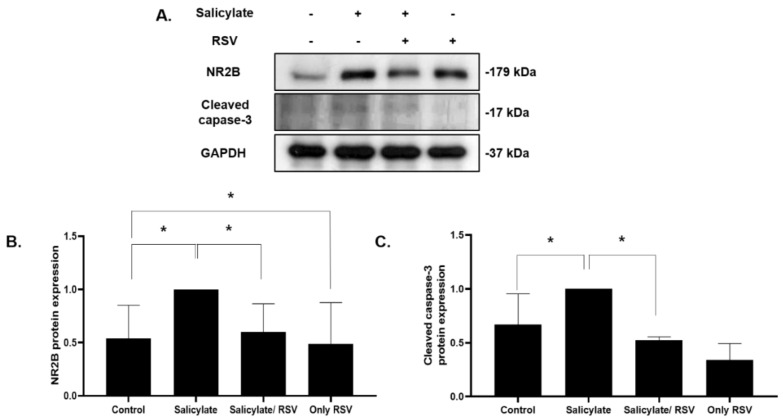
Effect of resveratrol (RSV) on the expression of NR2B and cleaved caspase-3 in rat cortical neuronal cells. Immunoblot analysis of the expression of NR2B and cleaved caspase-3 (**A**). Expression of NR2B (**B**) and cleaved caspase-3 (**C**) quantified using ImageJ (* *p* < 0.05, mean ± SD). GAPDH was used as the internal standard. NR2B: NMDA receptor subunit 2B.

We then confirmed the protective effects of RSV in the primary culture cortical neurons. Rat cortical neurons were isolated and cultured in conditioned media and subjected to an immunoblot analysis with antibodies specific for NR2B and the apoptotic protein cleaved caspase-3. The NR2B and cleaved caspase-3 expression was increased in the salicylate-treated cells; it decreased with the RSV pretreatment (Figure 4).

### 2.5. RSV Attenuates a Salicylate-Induced GPIAS Decrease in Rats

**Figure 5:**
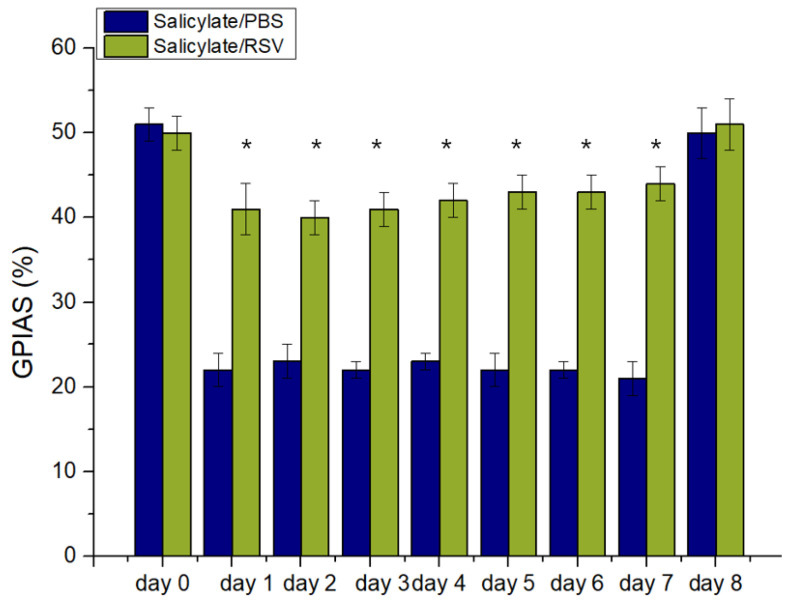
Resveratrol (RSV) attenuated a salicylate-induced decrease in gap-prepulse inhibition of the acoustic startle reflex (GPIAS) ratio. From day 1 to 7, RSV treatment protected against a salicylate-induced reduction of the GPIAS ratio in Sprague Dawley rats. * *p* < 0.05. PBS: phosphate-buffered saline.

As shown in Figure 5 there was no significant difference in the mean GPIAS value between the control group (52%) and the study group (50%) during the baseline session (day 0). However, there was a significant difference in the mean GPIAS values between the two groups during the drug administration; the mean GPIAS values decreased in the control group, but were attenuated by the RSV treatment in the study group. These results suggested that RSV protected against salicylate-induced tinnitus (*p* < 0.05) (Figure 5).

### 2.6. RSV Attenuates a Salicylate-Induced Elevation of the ABR Threshold in Rats

**Figure 6:**
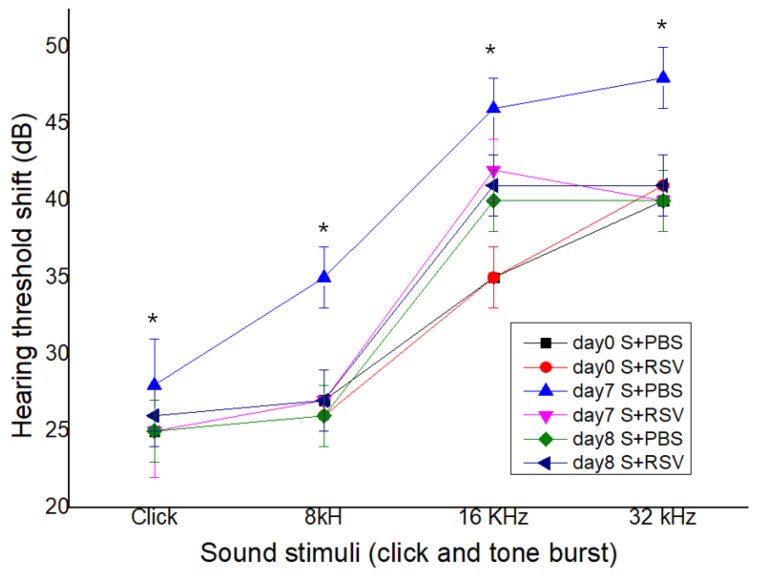
Resveratrol (RSV) attenuated a salicylate-induced elevation of auditory brainstem response (ABR) threshold in rats. RSV attenuated a salicylate-induced elevated ABR threshold shift except in the click group; salicylate injection given for seven consecutive days. * *p* < 0.05.

As shown in Figure 6, a temporary elevated ABR threshold at 8, 16, and 32 kHz was confirmed in the control group on day 7 compared with that before the treatment (day 0), but RSV attenuated the elevation of the ABR threshold on day 7 in the study group. The mean ABR threshold in both groups did not significantly differ between before the treatment and one day after the end of the treatment (*p* > 0.05) (Figure 6). These results suggested that RSV could potentially protect against a salicylate-induced threshold shift.

### 2.7. Spatial Expression of NR2B in the Auditory Cortex after Salicylate and RSV Treatments

**Figure 7:**
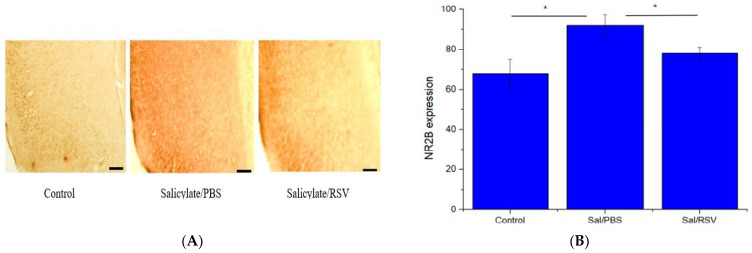
Resveratrol (RSV) attenuated NR2B expression in Sprague Dawley rats. NR2B expression in the control, salicylate/phosphate-buffered saline (PBS), and salicylate/RSV groups (**A**); scale bar: 100 µm, 100 × magnification. NR2B expression quantified using ImageJ. The salicylate/PBS group showed increased NR2B expression whereas RSV significantly attenuated NR2B expression (**B**); * *p* < 0.05. NR2B: NMDA receptor subunit 2B; PBS: phosphate-buffered saline.

As shown in Figure 7A, the salicylate-treated control group showed an increased NR2B expression in the auditory cortex whereas RSV significantly attenuated the NR2B expression (Figure 7B). In the group treated with salicylate and RSV, the NR2B expression decreased to the level of the vehicle-treated group.

## 3. Discussion

Salicylate is a widely prescribed drug due to its beneficial analgesic and anti-inflammatory properties [16]. However, its long-term, high-dose usage may lead to adverse effects such as tinnitus [17,18]. Salicylate abuse can stimulate the NMDA receptor and accelerate the intracellular Ca^2+^ influx, causing the excitotoxicity of the auditory nervous system, eventually leading to cellular damage [19]. Here, we examined the effects of salicylate on neuronal cells and identified that salicylate increased the expression of *NR2B* and its related genes, *TNFα* and *ARC*.

RSV is a well-known natural compound that has several beneficial effects [20,21]. RSV mitigates neurodegenerative disorders that involve Sirtuin 1 activation [22]. In a recently published paper, it was shown that the neuroprotective effect induced by RSV was mediated by a resistance to glutamate-induced excitotoxicity. The authors suggested that RSV could moderate glutamate release at the synaptic terminal by decreasing the protein kinase activity, which in turn relieved the voltage-gating Ca^2+^ channel activity [23]. Thus, RSV could be expected to have a protective effect on salicylate-induced neurotoxicity. In this study, we demonstrated that the increased expression of *NR2B* and its related genes induced by salicylate was reduced with an RSV pretreatment. At the cochlea level, the protective effect of RSV against cisplatin-induced ototoxicity in HEI-OC1 auditory cells has been reported [15]. RSV was decreased in cisplatin-induced ROS associated with resveratrol. Excessive ROS production is one of the most important causes of hair cell damage [24]. Non-steroidal anti-inflammatory drugs produce excessive ROS as a side effect of long-term use and can induce ototoxicity of the peripheral/central auditory neurons [25]. Salicylate is also known to increase ROS levels in patients if used for long periods and can eventually damage the cochlear spiral ganglion neurons [26]. Our in vitro experiments confirmed that salicylate increased the ROS levels, which were decreased by a pretreatment with RSV. CREB controls plasticity, neurogenesis, and survival in neurons [27]. Previous studies have shown that CREB was phosphorylated via the Ca^2+^/calmodulin-dependent protein kinase II (CaMKII) signaling pathway in the auditory cortex of mice with salicylate-induced tinnitus [28,29]. In accordance with these studies, we observed an increase in CREB phosphorylation in salicylate-treated neuronal cells, which decreased when the cells were pretreated with RSV. Son et al. [30] reported that glutamate at high concentrations induced neuronal cell death and ROS formation using HT22 neuronal cells. They observed that piceatannol reduced glutamate-induced cell death and ROS formation. RSV and piceatannol have been shown to have antioxidant and anti-apoptotic effects [31].

Short-term tinnitus develops shortly after a high-dose salicylate injection. The known mechanism of salicylate-induced tinnitus in animals is NMDA receptor stimulation [32]. The development of a GPIAS measurement has enabled the study of animal research in a tinnitus model.

In this study, we focused on 16 kHz for the GPIAS. According to the review paper of Galazyuk et al. [33], most salicylate studies were performed on rats for a tinnitus induction. It has been shown that 1–2 h after a systemic salicylate injection ranging in dosage from 150 to 400 mg/kg, rats exhibit GPIAS deficits typically around 16 kHz [32] and rarely at a wider range of frequencies [33]. Ralli et al. [34] reported that rats treated with salicylate alone (SAL) showed a significant reduction in GPIAS at 16 kHz, which was consistent with tinnitus-like behavior with a pitch near 16 kHz. These findings were confirmed by our previous paper [14].

High doses of salicylate cause hyperactivation in the hippocampus and the cerebral cortex (including the auditory cortex). The plasticity of these cortical excitatory neurons is involved in salicylate-induced tinnitus [6,34,35,36]. We observed that NR2B overexpression and excessive ROS production in salicylate-treated cells were reversed when they were pretreated with RSV. These beneficial effects of RSV were confirmed in the primary cortical neurons. Thus, these results suggest that RSV protects against cell excitotoxicity. Based on our results in a tinnitus animal model, RSV therapy could be proposed as an effective treatment for tinnitus.

## 4. Materials and Methods

### 4.1. Cell Culture and Treatment

SH-SY5Y cells were cultured in Dulbecco’s Modified Eagle’s Medium/F12 supplemented with 10% fetal bovine serum, L-glutamine, and 1% antibiotics and incubated in a humidified atmosphere of 5% CO_2_ at 37 °C. The cells were differentiated by a retinoic acid (1 µM) treatment in a 0.1% serum-supplemented medium for 2 days [37]. The differentiated cells were treated with 2 µM RSV in a 0.1% serum-supplemented medium for 12 h and then treated with 40 µg/mL salicylate in a 0.1% serum-supplemented medium for 8 h.

The cerebral cortex was dissected from Sprague Dawley rat pups at embryonic day 18 and prepared for the cell culture. The primary rat cortical neuron cells were seeded (0.5 × 10^6^ cells/well) on poly-L-lysine (150 µg/mL; Sigma-Aldrich)-coated 6-well plates. The cells were cultured in a growth medium containing Neurobasal A, 1X B27 supplement (Invitrogen), 100 units/mL penicillin, 0.1 mg/mL streptomycin, and 0.5 mM glutamine (Invitrogen) and incubated in a humidified incubator set at 37 °C supplied with 5% CO_2_. These cells were subjected to the same treatment method as that for the SH-SY5Y cells, except for the use of serum media.

### 4.2. Quantitative Polymerase Chain Reaction (qPCR)

The total RNA was extracted from the treated cells using RNAiso Plus (TAKARA, Tokyo, Japan). The cDNA was synthesized using PrimeScript II 1st Strand cDNA Synthesis kits (TAKARA) with the supplied buffer (0.2 μg random primers and 1 mM dNTPs). The PCR was performed using human and rat gene-specific primers specific for *NR2B*, tumor necrosis factor-alpha (*TNFα*), activity-regulated cytoskeleton-associated protein (*ARC*), and *β-actin* using the Power SYBR Green PCR Master Mix (Applied Biosystems, Carlsbad, CA, USA). The primers were synthesized by Genotech (Daejeon, Korea) and Integrated DNA Technologies Inc. (Coralville, IA, USA). Details about the primers are summarized in Table 1.

### 4.3. Immunocytochemistry Staining

SH-SY5Y cells were cultured on poly-D-lysine-coated coverslips (Thermo Fisher Scientific, Waltham, MA, USA) and differentiated into neuronal cells. Following the treatment, the cells were fixed with 4% paraformaldehyde and incubated with primary antibodies specific for NR2B (1:200; Santa-Cruz Biotechnology, CA, USA) and p-CREB (1:200; Santa Cruz Biotechnology) for 90 min at room temperature (RT) followed by incubation with Alexa 555-conjugated donkey anti-mouse IgG (1:500; Molecular Probes Inc., Eugene, OR, USA) and Alexa 488-conjugated donkey anti-rabbit IgG secondary antibodies (1:500; Molecular Probes Inc.), respectively, along with Hoechst 33342 (1:1000; Molecular Probes Inc.) in phosphate-buffered saline (PBS) for 90 min at RT. The cells were then mounted (ProLong Gold anti-fade reagent; Molecular Probes Inc.) and visualized under a Nikon Eclipse Ti2 fluorescence microscope (Nikon, Tokyo, Japan). The images were taken by a DS-Ri2 digital camera (Nikon).

### 4.4. Immunoblotting

The proteins were isolated from the cells using a radioimmunoprecipitation assay (RIPA) buffer along with a protease inhibitor cocktail, 2 mM phenylmethylsulphonyl fluoride (PMSF), and 1 mM sodium orthovanadate. The cell lysates were centrifuged at 16,000× *g* for 20 min at 4 °C and the protein was quantified. Equal amounts of proteins were resolved by SDS-PAGE using 6–15% resolving gels and electro-transferred onto a polyvinylidene difluoride (PVDF) membrane. The membrane was blocked with 5% normal horse serum (NHS) in TBS-T and then incubated overnight at 4 °C with primary antibodies specific for NR2B (1:200; Santa Cruz Biotechnology), p-CREB (1:500; Santa Cruz Biotechnology), CREB (1:500; Santa Cruz Biotechnology), cleaved caspase-3 (1:150; Merck Millipore, CA, USA), p53 (1:500; Santa Cruz Biotechnology), and GAPDH (1:1000; Santa Cruz Biotechnology) as well as horseradish peroxidase-conjugated anti-rabbit, anti-mouse, and anti-goat (Santa Cruz Biotechnology). The protein bands were visualized by enhanced chemiluminescence detection (GE Healthcare, Buckinghamshire, UK) and quantified using ImageJ software 1.53t, Bethesda, MD, USA). All the bands were normalized with GAPDH and p-CREB was normalized with CREB.

### 4.5. ROS Assay

The ROS levels were analyzed using 2′,7′-dichlorofluorescein diacetate (DCFH-DA). Briefly, the differentiated cells (approximately 70% confluence) were incubated with 2 μM RSV in 0.1% FBS for 12 h and then treated with 40 μg/mL salicylate for 8 h at 37 °C. The cells were then incubated with 20 μM DCFH-DA at 37 °C for 20 min. They were mounted and visualized under a Nikon Eclipse Ti2 fluorescence microscope. The ROS levels were measured using ImageJ software.

### 4.6. In Vivo Experiments

#### 4.6.1. Animals

**Figure 8:**
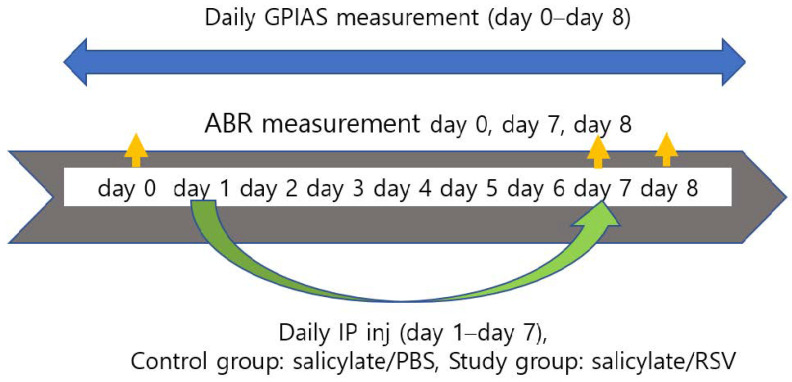
Schematic diagram of in vivo experimental procedure for investigating the tinnitus-attenuating effect of resveratrol in Sprague Dawley (SD) rats. Drugs were administered from day 1 to day 7 in the control and study groups (salicylate, RSV (resveratrol), and PBS (phosphate-buffered saline)). Gap-prepulse inhibition of acoustic startle reflex (GPIAS) was measured from day 1 to 8. Auditory brainstem response (ABR) thresholds were evaluated on day 0 as well as on days 7 and 8.

Twenty adult male Sprague Dawley rats (weighing 250–300 g) with normal eardrums and Preyer’s reflex were used in this study. This study was approved by the Institutional Animal Care and Use Committee (C IACUC-2020-S0019). The rats were randomly classified into two groups, a control group (*n* = 10, salicylate treatment group) and a study group (*n* = 10, salicylate and RSV co-treatment group). The ABR and GPIAS were evaluated in both groups. The control group received an intraperitoneal (IP) injection of sodium salicylate (Sigma-Aldrich, 400 mg·kg^−1^·day^−1^) combined with PBS (0.2 mL) whereas the study group received an IP injection of the same dose of sodium salicylate combined with RSV (Sigma, 250 mg·kg^−1^·day^−1^). The IP injections were administered daily for 7 days (Figure 8).

#### 4.6.2. GPIAS Measurement

The GPIAS measurement method used in this study to measure the startle response was similar to that reported in our previous study [32]. In brief, the GPIAS system consisted of a mesh cage with a vibration sensor, a noise box with an anechoic inner wall, an acoustic stimulator with a full-range loudspeaker (PM-5004 amplifier; Marantz, Kawasaki, Japan), a reference microphone (40 PH; GRAS, Holte, Denmark), a sensor signal acquisition hardware tool (PC and NI PCIe-6321; National Instruments, Austin, TX, USA), and LabVIEW-based custom graphical user interface (GUI) software. Each rat was subjected to a session comprising 15 gap-conditioned stimuli-evoked startle responses and 15 responses with no gap. Additionally, two types of acoustic stimuli (gap and no gap) were given to rats in a random order. The inter-stimulus interval (ISI) of these acoustic stimuli was randomized between 17 and 23 s to eliminate the habituation effect that might arise from a fixed startle-stimulus interval [32]. In addition, the rats were placed in the cage for about 2 min before the first session to acclimatize them to the measurement environment. The two types of acoustic stimuli mentioned above included a continuous narrowband background noise with a 1 kHz bandwidth, 16 kHz center frequency, and 60 dB sound pressure level (SPL) and a short high-level sound as a startle stimulus (broadband noise burst, 50 ms in length, 105 dB SPL) [32,38]. The central frequency of the narrowband background noise used in the stimulation condition followed the typical GPIAS measurement conditions for the salicylic acid-induced SD-rat tinnitus model used in several previous studies [33,39].

The gap-conditioned stimulus included a silence prepulse and a gap starting 100 ms before the startle stimulus and lasting for 50 ms [32]. The GPIAS value was defined as a percentage of the reduction in the gap-conditioned stimulus response relative to the magnitude of the no-gap-conditioned one [32].

#### 4.6.3. ABR Measurement

ABR was evaluated using a TDT system (Tucker-Davis Technologies, Miami, FL, USA) in an electrically sound-shielded sound-proof box. As shown in Figure 1, the measurement of the ABR thresholds was performed on day 0 (before the drug administration), day 7 (end of the administration), and day 8 (one day after the drug administration). The measurement method was similar to that used in our previous study [30]. In brief, subdermal needle electrodes were placed at the vertex (active) below the left pinna (reference) and the electrode was inserted under the right ear (ground). The stimuli consisted of click and tone bursts (8, 16, and 32 kHz) with reducing levels in the range of 10–90 dB of 5 dB intervals to determine the lowest intensity level. Each measurement point was recorded and averaged 1000 times. The stimuli were presented at a rate of 19 s and the acoustic stimuli were calibrated before the measurement of each group.

#### 4.6.4. Preparation of Free-Floating Sections

For the histological examination of their brain tissue, the rats were sacrificed 2 days after the last injection of each drug or vehicle. They were anesthetized and perfused with 4% paraformaldehyde (PFA) in PBS (pH 7.4). Their brains were immediately removed, stored in 4% (*w*/*v*) PFA in PBS for 2 days at 4 °C, suspended in 30% (*w*/*v*) sucrose for 4 days, and then embedded in an optimum cutting temperature compound (Miles Inc., Elkhart, IN, USA). Using a sliding microtome (SM2010R; Leica Microsystems, Wetzlar, Germany), the brain hemispheres were coronally sectioned for the auditory cortex at approximately 4.30–5.30 mm caudal to the bregma. Free-floating serial sections (30 μm thick) were collected into 10 PBS-filled wells.

#### 4.6.5. Immunohistochemistry

The brain samples were collected and fixed using 4% (*w*/*v*) PFA in PBS. The samples were immersed in 30% (*w*/*v*) sucrose for at least 4 days, following which they were sectioned into coronal slices (30 μm thick) using a frozen sliding microtome (SM2010R) and stored in PBS at 4 °C. To disable the intrinsic peroxidase activity, the sections were incubated in 0.3% (*v*/*v*) hydrogen peroxide in distilled water for 20 min. This was followed by 1 h of blocking with 5% (*v*/*v*) normal goat serum (Vector ABC Elite Kit; Vector Laboratories, Burlingame, CA, USA) in 0.3% (*v*/*v*) Triton X-100. The sections were then incubated with a rabbit anti-NMDAR2B antibody (1:200; Abcam, Cambridge, UK) diluted with an antibody dilution buffer (Invitrogen) at 4 °C overnight. After washing, the sections were reacted with biotinylated goat anti-rabbit IgG (Vector ABC Elite Kit; Vector Laboratories) for 1 h and washed again. The sections were then incubated for 1 h at RT with an avidin–biotin–peroxidase complex (Vector ABC Elite Kit; Vector Laboratories) according to the manufacturer’s instructions. After washing, the diaminobenzidine substrate (DAB kit; Vector Laboratories) was used as the chromogen to visualize the signal.

The immunoreactivity of NMDAR2B in the auditory cortex subregions was analyzed by measuring the intensity of the NMDAR2B-immunopositive reaction with ImageJ software. Coronal sections (30 μm thick) were selected approximately 2.5–3.6 mm posterior to the bregma in each brain and the intensities in the subregion were assessed. The intensity levels were expressed as a mean ± standard error (SE; *n* = 4 rats/group).

### 4.7. Statistical Analysis

An analysis of variance (ANOVA) with Tukey’s HSD post hoc test (three or more groups) was conducted using GraphPad Prism to determine the statistical significance. If the *p*-value was less than 0.05, it was considered to be statistically significant.

## 5. Conclusions

Our results provide evidence that RSV may reduce salicylate-induced tinnitus in rats. Further studies should determine the potential therapeutic effects of RSV in clinical settings.

## Figures and Tables

**Table 1:** Primers used for the quantitative polymerase chain reaction.

Gene	Forward Primer (5′→3′)	Reverse Primer (3′→5′)	Gene Accession
***Human β-actin***	ATCCGCAAAGACCTGTACGC	TCTTCATTGTGCTGGGTGCC	NM_001101
***Human NMDA (NR2B)***	GGAGAGGTGGTCATGAAGAG	CATTGCTGCGTGACACCATG	NM_000834.4
***Human TNFα***	GTTGTAGCAAACCCTCAAGCTG	CCAGCTGGTTATCTCTCAGCTC	NM_000594.3
***Human ARC***	ACAACAGGTCTCAAGGTTCCC	AGCCGACTCCTCTCTGTAGC	NM_015193.4
***Rat GAPDH***	CTGCCACTCAGAAGACTGTGG	TTCAGCTCTGGGATGACCTTG	NM_017008.4
***Rat NMDA (Nr2b)***	GGAGATGGAAGAACTGGAAGCTC	GACACCTGCCATATTGTCGATG	NM_012574.1
***Rat TNFα***	CCACCACGCTCTTCTGTCTAC	GATGATCTGAGTGTGAGGGTCTG	NM_012675.3
***Rat ARC***	GTCTGCTGCATAGAAGGAACCAG	AGGGTGCCCACCACATACTGA	NM_019361.1

## Data Availability

The data presented in this study are available on request from the corresponding author.
