# Correction: Song et al. Protective Effect of Resveratrol in an Experimental Model of Salicylate-Induced Tinnitus. *Int. J. Mol. Sci.* 2022, *23*, 14183

**DOI:** 10.3390/ijms24119460

**Published:** 2023-05-30

**Authors:** Anji Song, Gwang-Won Cho, Changjong Moon, Ilyong Park, Chul Ho Jang

**Affiliations:** 1 Department of Biology, College of Natural Science, Chosun University, Gwangju 61452, Republic of Korea; 2 BK21 FOUR Education Research Group for Age-Associated Disorder Control Technology, Department of Integrative Biological Science, Chosun University, Gwangju 61452, Republic of Korea; 3 Department of Veterinary Anatomy, College of Veterinary Medicine and BK21 FOUR Program, Chonnam Natioanal University, Gwanjgu 61186, Republic of Korea; 4 Department of Biomedical Engineering, College of Medicine, Dankook University, Cheonan 31116, Republic of Korea; 5 Department of Otolaryngology, Chonnam National University Medical School, Gwangju 61469, Republic of Korea

In the original publication [1], there was a mistake in Figure 2A as published. Accidentally, the same figure was used in Figures 2A and 3A. The corrected Figure 2A appears below. 


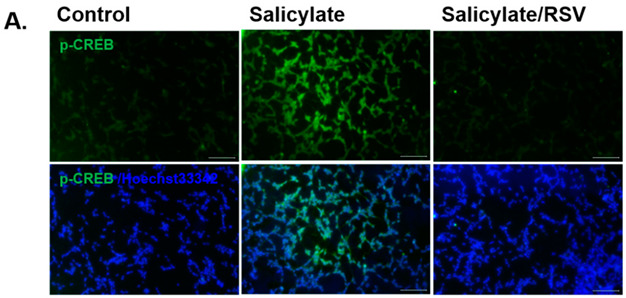


The authors apologize for any inconvenience caused and state that the scientific conclusions are unaffected. This correction was approved by the Academic Editor. The original article has been updated.
